# The Association Between Clinical Outcome and Expression of DNMT1, 3A, and 3B in Locally Advanced Laryngeal Carcinomas Treated by Definitive Radiotherapy

**DOI:** 10.3390/cancers17111741

**Published:** 2025-05-22

**Authors:** Karlijn van den Bovenkamp, Gyorgy B. Halmos, Lorian Slagter-Menkema, Boukje A. C. van Dijk, Shibo Yu, Johannes A. Langendijk, Bernard F. A. M. van der Laan, Ed Schuuring, Bert van der Vegt

**Affiliations:** 1 Department of Otorhinolaryngology/Head and Neck Surgery, University Medical Center Groningen, University of Groningen, 9723 GZ Groningen, The Netherlands; k.van.den.bovenkamp@umcg.nl (K.v.d.B.); g.b.halmos@umcg.nl (G.B.H.); l.menkema@umcg.nl (L.S.-M.); b.f.a.m.van.der.laan@rug.nl (B.F.A.M.v.d.L.); 2 Department of Pathology, University Medical Center Groningen, University of Groningen, 9723 GZ Groningen, The Netherlands; s.yu@umcg.nl (S.Y.); e.schuuring@umcg.nl (E.S.); 3 Department of Epidemiology, University Medical Center Groningen, University of Groningen, 9723 GZ Groningen, The Netherlands; b.a.c.van.dijk@umcg.nl; 4 Department of Research and Development, Comprehensive Cancer Organisation The Netherlands (IKNL), 3501 DB Utrecht, The Netherlands; 5 Department of Radiotherapy, University Medical Center Groningen, University of Groningen, 9723 GZ Groningen, The Netherlands; j.a.langendijk@umcg.nl; 6 Department of Otorhinolaryngology/Head and Neck Surgery, Haaglanden Medical Center, 2512 VA The Hague, The Netherlands

**Keywords:** laryngeal squamous cell carcinoma, recurrence, DNA methyltransferase, methylation, immunohistochemistry

## Abstract

**Objectives**: This study aimed to evaluate the prognostic significance of DNA methyltransferases (DNMTs) expression, including DNMT1, DNMT3A, and DNMT3B, in assessing the risk of locoregional recurrence after radiotherapy in patients with locally advanced laryngeal squamous cell carcinoma (LSCC), in order to optimize treatment decision making. **Methods**: A retrospective analysis was performed on pre-treatment biopsy tissues and clinical data from 58 patients with locally advanced LSCC (stages T3–T4, M0) treated with primary curative radiotherapy. DNMT expression was assessed through immunohistochemistry, and Cox regression analysis was applied to examine associations between methylation marker expression, demographic and clinical data, and both locoregional recurrence and disease-specific mortality. **Results**: Low expression of DNMT3A (*p* = 0.045) and the presence of locoregional lymph node metastases at diagnosis (N+-status) (*p* = 0.002) were associated with disease-specific mortality. Clinical N-status was also associated with locoregional recurrent disease after primary radiotherapy (*p* < 0.001). Expression of DNMT1 and DNMT3B, age, sex, and clinical T-status were not associated with locoregional recurrences or disease-specific mortality. **Conclusions**: Low expression of DNMT3A and the presence of regional lymph node metastases were independently associated with disease-specific mortality in patients with locally advanced LSCC treated primarily with definitive, curatively intended radiotherapy.

## Simple Summary

Locally advanced laryngeal cancer is challenging to treat, as it can return after primary treatments such as radiotherapy. This study examines the expression levels of certain proteins (DNMT1, DNMT3A, and DNMT3B) in tumor tissue, using immunohistochemistry to assess whether these protein levels are linked to the risk of cancer recurrence or mortality. By studying pre-treatment biopsy samples, we aimed to identify markers that could improve the estimation of treatment outcomes. Our results show that lower levels of the protein DNMT3A and the presence of lymph node involvement were associated with a higher risk of mortality. These findings may support more personalized treatment strategies and deepen the understanding of how these proteins relate to cancer progression.

## 1. Introduction

Of the approximately 209,000 patients that are annually diagnosed globally with laryngeal squamous cell carcinomas (LSCCs) [1], around 30% have locally advanced disease [2]. Treatment of locally advanced LSCC consists of radiotherapy with or without systemic treatment, surgery, or a combination of these modalities. If possible, organ-preserving strategies are preferred, as a higher quality of life with similar survival compared to more extensive surgery can be achieved [3]. Despite improvements in treatment strategies, locoregional recurrences still develop in around 25% of the patients [4,5]. In case of recurrent disease after radiotherapy with or without systemic treatment, salvage surgery is sometimes possible, but both oncological and functional outcome are worse compared to primary surgery [6,7,8]. Moreover, impaired wound healing can be expected after salvage surgery, which leads to further morbidity [9]. Prognostic factors to predict outcome after (chemo)radiation are lacking, which makes it difficult to optimize treatment decision making for individual patients.

**Figure 1:**
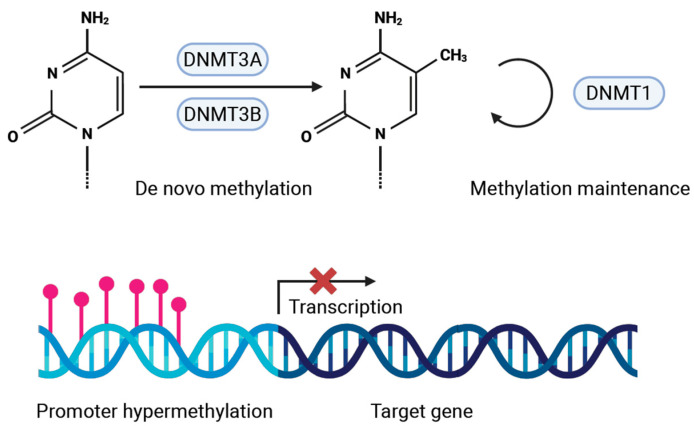
Role of DNA methyltransferases in promoter hypermethylation and transcriptional silencing. Created in BioRender. van den Bovenkamp, K. (2025) https://BioRender.com/hhbl4f0 (accessed on 14 May 2025).

DNA methylation is an epigenetic mechanism that plays a key role in tumorigenesis, by changing the methylation status of tumor-suppressor genes involved in cell cycle regulation, apoptosis, and DNA repair [10,11,12,13,14]. DNA methyltransferases are a family of proteins involved in DNA methylation, and include DNMT1, DNMT3A, and DNMT3B. The primary function of DNMT1 is maintaining CpG methylation status during cell division (maintaining the cancer stem cell status), while DNMT3A and DNMT3B are predominantly involved in de novo methylation [15,16] (Figure 1).

Aberrant DNA methylation patterns have been observed in a variety of tumors [17,18], including in HNSCC [10,19,20]. Alteration in DNA methylation plays a role in head and neck tumor radioresistance [21], with aberrant hypomethylation or hypermethylation leading to radioresistance depending on the function of the genes [22,23]. DNMT expression patterns may be associated with outcome after radiotherapy for LSCC.

The aim of this study was to investigate associations between methylation markers (DNMT1, DNMT3A, and DNMT3B), locoregional control, and disease-specific survival in a well-defined homogeneous cohort of patients with locally advanced (T3–T4, M0) LSCC treated with definitive, curatively intended radiotherapy alone.

## 2. Materials and Methods

### 2.1. Patients

The retrospective selection of patients and samples was performed as reported previously [24]. Briefly, patients were included in the study if they met the criteria of having histologically confirmed T3 or T4 laryngeal squamous cell carcinoma without evidence of distant metastasis, as defined by the American Joint Committee on Cancer, and if they received definitive curatively intended radiotherapy without systemic or surgical treatment at the University Medical Center Groningen between December 1992 and February 2015. Inclusion depended on the availability of sufficient pretreatment tumor material at the Department of Pathology of the University Medical Center Groningen. Fifty-eight patients met all inclusion criteria.

The Local Ethics Review Board Pathology non-WMO studies (LTc Pathology) at the University Medical Center Groningen determined that this study falls outside the scope of the Medical Research Involving Human Subjects Act (WMO) (registration number: 202100893).

### 2.2. Treatment and Follow-Up

Treatment and follow-up were as described before [24]. Up to 2007, curative three-dimensional conformal radiotherapy (3D-CRT) was used with 23 × 2 Gy on the indicated elective neck levels and a sequential boost on the primary tumor and lymph node metastases to a total dose of 70 Gy. Since 2007, intensity modulated radiotherapy (IMRT) with a simultaneous integrated boost (SIB) was applied, with 35 × 2 Gy on the primary tumor and lymph node metastases, and generally elective irradiation of the neck to a total dose of 54.25 Gy, in fractions of 1.55 Gy.

Follow-up was in accordance with the Dutch Head and Neck Society guidelines [25] and included flexible endoscopy and physical examination of the neck every 3 months during the first 2 years and every 6 months thereafter, up to a total of 5 years after completion of radiotherapy. Treatment response evaluation using CT imaging of the head and neck region was performed around 8 weeks after the last day of radiotherapy. When residual or recurrent disease was suspected, endoscopic evaluation with histological biopsy was performed under general anaesthesia. In case of residual disease, salvage treatment (mostly consisting of total laryngectomy) was given when feasible.

### 2.3. Analysis of Publicly Available Data

For *The Cancer Genome Atlas* (TCGA) cohort, RNA-sequencing data, pathological characteristics, and survival information were downloaded through cBioPortal (https://www.cbioportal.org/ accessed on 1 January 2025) and UCSC Xena (https://xenabrowser.net/datapages/ accessed on 1 January 2025) databases. After applying selection criteria, we identified 48 T3–T4 laryngeal cancer patients, all of whom underwent radiotherapy. DNMT1, DNMT3A, and DNMT3B mRNA expression levels were compared to outcome.

### 2.4. Immunohistochemistry and Scoring

Three μm thick paraffin sections were deparaffinised using xylene twice for 10 min and rehydrated through a series of decreasing ethanol dilutions and phosphate buffered saline (PBS). Antigen retrieval was performed by boiling slides in preheated 10 mM citrate buffer, pH 6.0 (DNMT1 and DMNMT3A) or 0.1 M Tris/HCl, pH 9.0 (DNMT3B) for 15 min. To block endogenous peroxidase activity, 0.3% hydrogen peroxide was applied for 30 min at room temperature. The slides were incubated for 1 h with mouse monoclonal antibodies (DNMT1 (1:100, Clone 60B1220.1, Novus Biologicals, Centennial, CO, USA), DNMT3A (1:200, Clone 64B1446, Novus Biologicals) and DNMT3B (1:100, clone 52A1018, Novus Biologicals)) and subsequently incubated for 30 min with EnVision, a peroxidase-conjugated polymer backbone. The slides were developed with di-amino benzidine (DAB) chromogen solution, followed by a haematoxylin counterstaining. Scoring of immunohistochemical staining was performed by two independent observers (KvdB and LSM) who were blinded for clinical outcome. Discordant cases were discussed and resolved by both observers and a dedicated head and neck pathologist (BvdV) during a consensus meeting.

The percentage of positively staining tumor cells was scored, as was the intensity of the staining. Nucleus and cytoplasm were scored separately.

**Figure 2:**
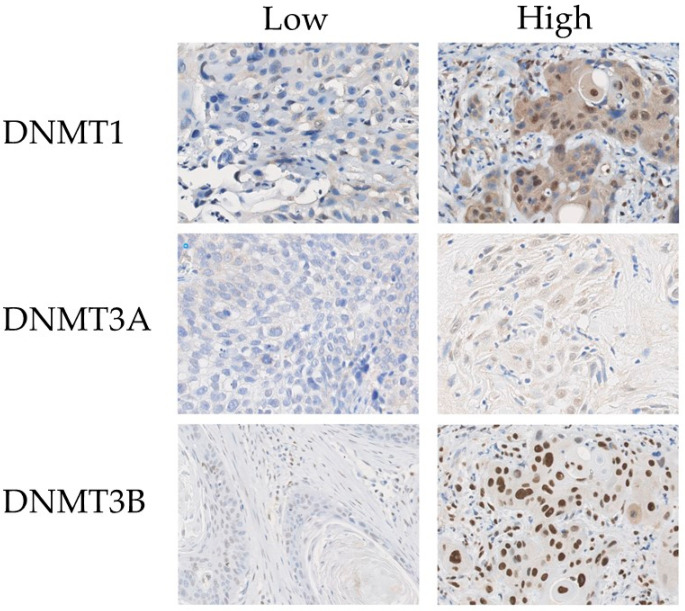
Immunohistochemical expression of DNMT1, DNMT3A, and DNMT3B (shown at 40× magnification).

For DNMT1, expression was considered high when ≥80% of the tumor cells showed a nuclear staining stronger than the cytoplasmic background, as described before [20]. For DNMT3A and DNMT3B, the median percentage of tumor cells with nuclear staining was used as cut-off value, which was 70% and 85%, respectively. (Figure 2).

### 2.5. Statistical Analysis

For the TCGA data, the “survival” and “survminer” packages in R (version 4.4.2) were used to perform multivariable survival analyses.

The Statistical Package for Social Sciences (version 28 for Windows, Armonk, NY, USA: IBM Corp.) was used for statistical analysis of the UMCG cohort. Age was dichotomized with a cut-off value of 65 years of age, and N-status was dichotomized based on the presence of regional lymph node metastases at diagnosis (N0 vs. N+). Locoregional recurrence was defined as tumor recurrence at the primary tumor site or in the cervical lymph nodes within 2 years after the last day of treatment. Disease-specific mortality was defined as death as a consequence of LSCC.

Time calculations were performed using the date of completion of radiotherapy as a starting point. The endpoints for locoregional recurrences were diagnosis of locoregional recurrence, last follow-up, or death. Patients who were lost to follow-up or deceased were censored. For disease-specific mortality, the endpoints included death resulting from LSCC, death due to other causes, or the last follow-up of the patient. Patients who were lost to follow-up or deceased due to other causes were censored.

Pearson’s correlation test was used to evaluate the correlation between expression percentages of DNMT1, DNMT3A, and DNMT3B. Chi-Square test, Fisher’s exact test, and Mann–Whitney U test were used to evaluate the association between clinical parameters and expression of DNMT1, DNMT3A, and DNMT3B. Univariable and multivariable Cox regression analysis was applied to evaluate the association of methylation markers and patient and tumor characteristics with the occurrence of locoregional recurrences and disease-specific mortality.

Besides our biomarkers of interest, variables associated with locoregional control or disease-specific mortality on univariable analysis (*p* < 0.05) were included in the multivariable analysis. If the Pearson’s correlation test indicates a correlation among the biomarkers of interest, they will undergo separate testing to ensure an adequately sized group. The Cox regression analysis fulfilled the proportional hazard assumption, as assessed through LML plots. A *p*-value < 0.05 was considered statistically significant for all analyses.

## 3. Results

### 3.1. Analysis on Publicly Available Data

DNMT3A mRNA expression in the TCGA cohort showed a trend toward longer overall survival and disease-specific survival in multivariable Cox regression analysis after adjusting for age, sex, N-status, and DNMT1 and DNMT3B mRNA expression. However, these trends did not reach statistical significance (OS: HR = 0.52, 95% CI = 0.22–1.25, *p* = 0.14; DSS: HR = 0.41, 95% CI = 0.08–2.21, *p* = 0.3). Based on these findings, we explored DNMT1, DNMT3A, and DNMT3B expression in our own cohort.

### 3.2. Demographic and Clinical Data

The demographic and clinical data of the study population are listed in Table 1. Fifty-eight patients were included, of which 51 were male (88%) and 7 female (12%), with a median age of 68.8 years (range 41.7–84.9). Sixty percent of the patients had a clinical T3-classification at presentation, and most of them had no cervical lymph node metastases (74%). The median follow-up was 51.9 months. Sixteen patients (28%) developed a locoregional recurrence, 10 patients (17%) died from disease, and 20 patients (34%) died from other causes.

No significant associations were found between clinical characteristics and DNMT1, DNMT3A, or DNMT3B (Supplementary Table S1).

Pearson’s correlation test showed a significant correlation between expression percentages of DNMT1 and DNMT3b (*p* < 0.001), and DNMT3A and DNMT3B (*p* < 0.001). This significant relation remained when the expression percentages were dichotomized and tested using the Chi-Square test (*p* = 0.024 and *p* < 0.001, respectively). No significant relation was found between expression percentages of DNMT1 and DNMT3A. Due to this correlation, it was decided to independently assess the biomarkers of interest in the multivariable analysis.

### 3.3. Locoregional Recurrence

In univariable cox regression analysis, low expression of DNMT3A and regional lymph node metastases (N+) were significantly related with the occurrence of locoregional recurrent disease (*p* = 0.031 and *p* < 0.001, respectively) (Table 2). Expression of DNMT1 and DNMT3B, as well as sex, age, and T-status, was not related to locoregional recurrences (Table 2).

In multivariable analysis, N-status was found to be the only independent predictor for locoregional recurrent disease (*p* < 0.001) (Table 3).

### 3.4. Disease-Specific Mortality

As shown in Table 2, low expression of DNMT3A, low expression of DNMT3B, and locoregional lymph node metastases at presentation (N+-status) were significant significantly associated with disease-specific mortality in univariable Cox regression analysis (*p* = 0.029, *p* = 0.047, *p* = 0.001, respectively).

When corrected for N-status in multivariable analysis (Table 4), low expression of DNMT3A remained significantly related to disease-specific mortality (*p* = 0.045). The expression of DNMT3B was not independently related to disease-specific mortality when corrected for N-status (*p* = 0.122) (Table 5).

## 4. Discussion

In this study, the associations between methylation markers (DNMT1, DNMT3A, and DNMT3B) and both locoregional recurrent disease and disease-specific mortality in patients with locally advanced LSCC treated with definitive, curatively intended radiotherapy alone was investigated. The objective was to contribute insights that could aid in the identification of patients who might derive optimal benefits from primary radiotherapy or necessitate consideration of alternative primary treatment modalities. Within this well-defined and homogeneous patient cohort, we observed that the low expression of DNMT3A and presence of locoregional lymph node metastases were associated with disease-specific mortality, while N-status was a significant factor associated with locoregional recurrent disease after primary radiotherapy. The role of DNMT3B remains uncertain, as its association with clinical outcomes observed in univariable analysis did not remain significant in multivariable analysis. This may be due to the limited sample size, or it could indicate that DNMT3B expression alone has limited clinical relevance in this context. Further studies with larger cohorts are needed to clarify the relevance of DNMT3B expression in this patient group.

Overexpression of DNMT3A was found in oral squamous cell carcinoma [26,27,28] and hepatocarcinogenesis [29], and increased expression of all three DNMTs was found in gastric cancer compared to normal tissue [30,31]. It is important to note that these studies did not investigate the response to radiotherapy but focused on expression levels relative to normal tissue. The only previous study investigating the influence of DNMT3A and DNMT3B expression on radiosensitivity found a lower radiosensitivity in embryonal rhabdomyosarcoma cells in case of overexpression [32]. However, this was found in a cell line model and not in clinical samples. In our study, low expression of DNMT3A was associated with disease-specific mortality in patients with LSCC primarily treated by radiotherapy. DNMT3A has a role in DNA damage repair. DNA repair mechanisms play a critical role in fixing DNA lesions caused by various factors, including radiation. When the DNA repair system is compromised, the tumor cells may have a diminished capacity to effectively repair radiation-induced DNA damage. Tumors harboring DNMT3A mutations may have adapted to this situation by activating alternative mechanisms to manage DNA damage, resulting in reduced sensitivity to radiotherapy. In the univariable analysis of our study, low expression of DNMT3A was associated with locoregional recurrences and showed a trend in multivariable analysis. Future larger studies could explore whether DNMT3A could serve as a biomarker for patient stratification, guiding treatment decisions, and whether DNMT3A inhibitors could be used to sensitize tumors to radiotherapy in advanced LSCC.

Increased expression of DNMT1, DNMT3A, and DNMT3B was found in various cancers such as breast cancer, pancreatic cancer, lung cancer, hematological malignancies, and head and neck squamous cell carcinoma [28,33,34]. Since increased expression of DNMTs have been associated with radioresistance in various cancers, DNMT-inhibitors could possibly enhance locoregional control after radiotherapy. Currently, numerous DNMT inhibitors are being developed, primarily categorized into nucleoside and non-nucleoside types based on their chemical structure. Nucleoside DNMT inhibitors are cytosine analogs that can either be incorporated into DNA sequences as triphosphates, thereby inhibiting DNA replication, or bind covalently to DNMT, leading to their degradation. The DNMT-inhibitors 5-azacitidine (5-AC) and 5-aza-2’-deoxycytidine (DAC) are FDA-approved for the treatment of hematological malignancies and myelodysplastic syndrome. The utility of DNMT inhibitors in solid tumors is emerging, but several pharmacokinetic challenges, such as high toxicity and low biostability, must be overcome before these inhibitors can be used in clinical practice. There have been a few studies investigating the effects of DNMT-inhibitors on solid human cancer cell lines treated with radiotherapy [28,35,36]. In lung cancer and glioblastoma cells, DNMT inhibitors Psammaplin A, 5-aza-2’-deoxycytidine, and zebularine enhanced radiosensitivity [35]. The novel phthalimido alkanamide derivative MA-17 showed radiosensitizing properties in human lung cancer cell cultures [36]. In oral squamous cell carcinoma cell lines, Zebularine demethylated DNA previously methylated by DNMTs and sensitized tumor cells for chemo and radiotherapy [28]. There are no studies evaluating the effect of DNMT inhibitors in patients with LSCC.

In the present cohort, no comparison of the expression profiles of DNMTs in normal laryngeal tissues and those in locally advanced LSCC was made, making it impossible to draw definitive conclusions about the potential efficacy of DNMT inhibitors as a therapeutic option for these patients. Therefore, it would be interesting to investigate the expression profiles of DNMT1, DNMT3A, and DNMT3B in both normal laryngeal tissue and (different stages of) LSCC. Additionally, it would be interesting to investigate the radiosensitizing ability of DNMT-inhibitors in patients with LSCC and overexpression of DNMTs.

The strength of this study is the well-defined homogeneous group of patients. Furthermore, it is the first study investigating expression of DNMT3A and DNMT3B in patients with laryngeal cancer, and it is the first study investigating DNMT1 in locally advanced LSCC. Nevertheless, inherent limitations, such as the retrospective study design and the constrained sample size, attributed to the specific characteristics of the patient cohort, necessitate careful consideration in extrapolating the findings of this study. Further (multi-institutional) studies are needed to validate our findings in larger patient series, and to explicate the prognostic role of DNMT3A and DNMT3B. 

This study primarily relied on pre-treatment biopsy samples, which may not fully capture the tumor’s heterogeneity or the potential changes in DNA methyltransferase expression during or after treatment. While pre-treatment samples are routinely used to guide therapeutic decisions, future research could benefit from incorporating both pre- and post-treatment samples to provide a more comprehensive understanding of the dynamic role of DNMT expression in treatment outcomes.

## 5. Conclusions

Low expression of DNMT3A and positive N-status were associated with disease-specific mortality in patients with advanced stage laryngeal squamous cell carcinoma primarily treated with radiotherapy. This finding should be confirmed in a larger study before we can use the expression of DNMT3A in LSCC treatment selection.

## Figures and Tables

**Table 1:** Demographic and clinical data of patients (n = 58).

Characteristic	Number of Patients (Row %)
Total N	58 (100)
Age, y	
Median	68.8
Range	41.7–84.9
Age, y	
<65	24 (41)
≥65	34 (59)
Sex	
Male	51 (88)
Female	7 (12)
Clinical T-classification	
T3	35 (60)
T4	23 (40)
Clinical N-classification	
N0	43 (74)
N1	10 (17)
N2	3 (5)
N3	2 (4)
Follow-up, mo	
Median	51.9
Range	1.9–145.9
Locoregional recurrence	
Yes	16 (28)
No	42 (72)
Disease-specific mortality	
Yes	10 (17)
No	48 (83)

Abbreviations: N, number of patients; y, years; mo, months.

**Table 2:** Univariable Cox regression analysis of immunohistochemical, demographic, and clinical data in relation with locoregional recurrence and disease-specific mortality (significant values are shown in bold).

Characteristic		Locoregional Recurrence		Disease-Specific Mortality	
		HR (95% CI)	*p*	HR (95% CI)	*p*
DNMT1	<80%	1	0.766	1	0.228
	≥80%	0.86 (0.31–2.36)		0.39 (0.08–1.82)	
DNMT3A	<70%	1	**0.031**	1	**0.029**
	≥70%	0.29 (0.09–0.89)		0.10 (0.01–0.80)	
DNMT3B	<85%	1	0.282	1	**0.047**
	≥85%	0.58 (0.22–1.56)		0.25 (0.07–0.98)	
Sex	Female	1	0.455	1	0.081
	Male	0.62 (0.18–2.18)		0.30 (0.08–1.16)	
Age, y	<65	1	0.402	1	0.606
	≥65	0.66 (0.25–1.75)		0.72 (0.21–2.49)	
T-status	T3	1	0.344	1	0.502
	T4	1.61 (0.60–4.28)		1.53 (0.44–5.30)	
N-status	N0	1	**<** **0.001**	1	**0.001**
	N+	6.46 (2.38–17.51)		9.94 (2.56–38.64)	

Abbreviations: HR, hazard ratio; 95% CI, 95% confidence interval; y, years.

**Table 3:** Cox regression analysis of DNMT3A and N-status in relation with locoregional recurrence (significant values are shown in bold).

Characteristic		Locoregional Recurrence	
		HR (95% CI)	*p*
DNMT3A	<70%	1	0.076
	≥70%	0.36 (0.11–1.11)	
N-status	N0	1	**<0.001**
	N+	5.63 (2.06–15.39)	

Abbreviations: HR, hazard ratio; 95% CI, 95% confidence interval.

**Table 4:** Cox regression analysis of DNMT3A and N-status in relation with disease-specific mortality (significant values are shown in bold).

Characteristic		Disease-Specific Mortality	
		HR (95% CI)	*p*
DNMT3A	<70%	1	**0.045**
	>70%	0.12 (0.02–0.96)	
N-status	N0	1	**0.002**
	N+	8.70 (2.21–34.22)	

Abbreviations: HR, hazard ratio; 95% CI, 95% confidence interval.

**Table 5:** Cox regression analysis of DNMT3B and N-status in relation with disease-specific mortality (significant values are shown in bold).

Characteristic		Disease-Specific Mortality	
		HR (95% CI)	*p*
DNMT3B	<85%	1	0.122
	≥85%	0.34 (0.09–1.34)	
N-status	N0	1	**0.002**
	N+	8.46 (2.15–33.32)	

Abbreviations: HR, hazard ratio; 95% CI, 95% confidence interval.

## Data Availability

The data that support the findings of this study are available upon request from the corresponding author.

## Supplementary Materials

The following supporting information can be downloaded at: https://www.mdpi.com/article/10.3390/cancers17111741/s1, Table S1: Associations between clinical characteristics and DNMT1, DNMT3A or DNMT3B.
