# Supplementary material for: The Association Between Clinical Outcome and Expression of DNMT1, 3A, and 3B in Locally Advanced Laryngeal Carcinomas Treated by Definitive Radiotherapy
Source: Cancers (Basel). 2025 May 22;17(11):1741. doi: 10.3390/cancers17111741 (PMC12153894; doi:10.3390/cancers17111741)
Supplement: Supplementary file 1 [file cancers-17-01741-s001.zip › cancers-3573804-supplementary.pdf]

Supplementary Table 1. Associations between clinical characteristics and DNMT1, DNMT3A or DNMT3B.

	DNMT1 p-value	DNMT3a p-value	DNMT3b p-value
Age (continuous variable)	0.499	0.243	0.628
Age (<65 years vs ≥65 years)	0.188	0.286	0.586
Sex	0.692	0.701	1.000
T-status	0.785	1.000	0.785
N-status (N0 vs N+)	0.760	0.373	0.235
